# Low-Intensity Transcranial Ultrasound Stimulation: Mechanisms of Action and Rationale for Future Applications in Movement Disorders

**DOI:** 10.3390/brainsci12050611

**Published:** 2022-05-07

**Authors:** Andrea Guerra, Matteo Bologna

**Affiliations:** 1 IRCCS Neuromed, 86077 Pozzilli, Italy; andrea.guerra@uniroma1.it; 2 Department of Human Neurosciences, Sapienza University of Rome, 00185 Rome, Italy

**Keywords:** transcranial ultrasound stimulation, TUS, movement disorders, Parkinson’s disease, essential tremor, dystonia, motor system, neuromodulation

## Abstract

Low-intensity transcranial ultrasound stimulation (TUS) is a novel non-invasive brain stimulation technique that uses acoustic energy to induce changes in neuronal activity. However, although low-intensity TUS is a promising neuromodulation tool, it has been poorly studied as compared to other methods, i.e., transcranial magnetic and electrical stimulation. In this article, we first focus on experimental studies in animals and humans aimed at explaining its mechanisms of action. We then highlight possible applications of TUS in movement disorders, particularly in patients with parkinsonism, dystonia, and tremor. Finally, we highlight the knowledge gaps and possible limitations that currently limit potential TUS applications in movement disorders. Clarifying the potential role of TUS in movement disorders may further promote studies with therapeutic perspectives in this field.

## 1. Introduction

Transcranial ultrasound stimulation (TUS) is a novel non-invasive brain stimulation (NIBS) tool that uses acoustic energy delivered through the intact skull to induce changes in neuronal activity [1,2]. The first human study dates back to 2013, when the TUS delivered on the frontal cortex was demonstrated to be able to improve pain and mood in patients with chronic pain [3]. In less than 10 years, significant effects of TUS on motor and somatosensory cortices have been reported using neurophysiological, neuroimaging, and behavioral outcome measures [2,4]. In addition, there was evidence that TUS can modulate the activity of deep brain areas, including the thalamus and brainstem nuclei [5,6,7]. In vitro, animal, and modeling studies suggest that the mechanisms of action of TUS depend on the biophysical effects produced by the acoustic energy of the ultrasound beam delivered to tissues. These effects include mechanical force resulting in the deformation/cavitation of the neuronal membrane and increased temperature in the target area, which partially absorbs ultrasound waves as they pass [1,2,8]. Membrane deformation and the formation of pores lead to capacitance changes or even cell membrane rupture, which in turn result in altered electrochemical cell properties and the activation of mechanosensitive calcium- and voltage-gated ion channels [9,10,11]. Similarly, the ultrasound-related temperature increase can transiently modulate neural activity by modifying the conductance of thermosensitive ion channels and altering membrane properties [12,13]. Notably, the sensitivity of different channels to mechanical forces or temperature may differ, and channel distribution may also significantly vary across cell types and brain regions. TUS effects may thus differ according to the brain area stimulated. Moreover, TUS may determine significantly variable effects, ranging from neural activity excitation to inhibition, depending on the stimulation parameters used [2,4,8,14]. The fundamental frequency of stimulation is crucial to the spatial targeting of brain areas and influences the attenuation of ultrasound by the skull. Indeed, increasing ultrasound frequency narrows the stimulation focus but results in greater transcranial attenuation and scattering [15]. For this reason, human TUS studies commonly adopt fundamental frequencies <1000 kHz. Moreover, TUS can be delivered with a continuous or pulsed sonication paradigm. Other stimulation parameters are the pulse repetition frequency (PRF), reflecting the pulse rate delivered, the duty cycle (DC), i.e., the proportion of time between the starting point of two consecutive sonications covered by the pulse duration, and the sonication duration (SD), referring to the total time from the onset of the first pulse to the termination of the last pulse. Finally, a very relevant TUS parameter is intensity, which can be measured as spatial-peak temporal average (i.e., average intensity during the entire sonication duration —I_SPTA_) and spatial-peak pulse average (i.e., average intensity of a single pulse—I_SPPA_). Importantly, high-intensity focused ultrasound (FUS) (I_SPPA_ > 200 W/cm^2^) induces permanent lesions in the brain through coagulation of cellular proteins and thermal ablation [16,17]. The rationale for the use of FUS for therapeutic purposes in neurological disorders is based on previous evidence obtained in studies based on lesional surgery and will not be further discussed in this paper. In this review, we will instead focus of low-intensity TUS (I_SPPA_ < 100 W/cm^2^), which is ideal for safe neuromodulation purposes [18]. Low-intensity TUS may either be delivered through commercially available diagnostic ultrasound imaging devices (i.e., unfocused TUS) or through dedicated systems that allow a more focused ultrasound beam (focused TUS—fTUS). Importantly, despite unfocused and fTUS acting through the same biophysical mechanisms, their intrinsic differences may play a role in determining different outcomes. fTUS implies the stimulation of a smaller brain volume, thus likely activating intracortical neural circuits in a more selective manner. Conversely, unfocused TUS may lead to a more widespread activation of neurons and fibers originating from the targeted area [2,4]. In addition, the sonication pattern is usually continuous for unfocused TUS and pulsed for fTUS, the latter more closely resembling repetitive TMS protocols able to induce plasticity effects [19,20,21].

Compared to other techniques, i.e., transcranial magnetic stimulation (TMS) and transcranial electrical stimulation (TES), the most significant advantage of low-intensity TUS is the depth penetration [4]. Stimulation can reach subcortical regions that are not easily accessible by TMS or TES. To date, despite the potential of low-intensity TUS as a novel NIBS tool, only a few research studies have been performed on healthy animal models or human subjects. Studies on animal models have been performed on a wide spectrum of neurological diseases, including dementia, epilepsy, and stroke [8,14]. In humans, to date only three pilot studies have been performed in Alzheimer’s disease [22,23,24] and two in epilepsy [25,26], and one case report involved a patient with a post-traumatic disorder of consciousness [7]. Concerning its use in movement disorders, low-intensity TUS has only been tested in a few parkinsonian animal models [27,28,29,30,31], though no study has been conducted in humans. Investigating low-intensity TUS effects in patients with movement disorders would be very useful for a deeper understanding of pathophysiological mechanisms and in order to develop novel therapeutic approaches. Low-intensity TUS could be effectively used in this context by leveraging existing knowledge. Along with changes in the basal ganglia-thalamo-cortical circuits in parkinsonism and dystonia pathophysiology, neurophysiological studies in these conditions have provided evidence of additional abnormalities at the cortical level, particularly in the primary motor cortex (M1), brainstem, and spinal cord circuits, including reduced inhibition, maladaptive plasticity, and altered sensory processing [32,33,34]. More recently, the role of the cerebellum and its interactions with the basal ganglia and cortical areas has been highlighted in both parkinsonism and dystonia pathophysiology [35,36]. Interestingly, in addition to the prominent role of the cerebellum and its major thalamic recipient (i.e., the ventral intermediate nucleus—Vim) in the pathophysiology of action tremor, abnormal interactions between the cerebellum and basal ganglia have also been proposed as a major mechanism in the pathophysiology of rest (parkinsonian) tremor [37,38].

In this perspective article, we first briefly describe the neurophysiological effects of TUS in healthy animals and humans in relation to the stimulated brain site. Based on these mechanisms and available TUS experimental data on animal models, we then discuss the possible rationale for using TUS in movement disorders and propose possible approaches for future studies in this research field.

## 2. TUS Effects in Healthy Animals and Humans

Concerning the neurophysiological effects of low-intensity TUS on cortical areas, the majority of the available studies have focused on motor areas, including M1 and non-primary motor areas, and the somatosensory cortex (S1). Investigations on possible TUS effects on other cortical areas are still limited. To the best of our knowledge, M1 studies in animals demonstrated excitatory effects, including the induction of subtle electromyography (EMG) responses and overt movements [39,40,41,42,43,44], primarily reflecting the intensity and duration of low-intensity TUS [45]. Additional mechanisms of action of low-intensity TUS of M1 include local field potential (LFP) changes with increased frequency of cortical spikes [39], cortical gamma power enhancement [46] in the stimulated area, increased phase synchronization between cortical LFP and EMG activity in various frequency bands [47], and increased cortical blood flow, which was linearly coupled with EMG motor response amplitude [48]. In humans, low-intensity TUS of M1 did not elicit motor responses per se [49,50,51,52,53]. However, in line with animal data, it has been reported that low-intensity fTUS of M1 during a cued finger-tapping task increased the functional magnetic resonance imaging (MRI) activation volume of the thumb representation [54]. It has also been observed that low-intensity unfocused TUS of M1 induces short-lasting motor evoked potential (MEP) amplitude changes reflecting increased corticospinal excitability [49]. However, opposite results were found when delivering TMS stimuli during low-intensity fTUS of M1. For example, concurrent single-pulse TMS and low-intensity fTUS led to M1 inhibition, as evidenced by decreased MEP amplitude and intracortical facilitation (ICF), a measure that specifically reflects facilitatory circuits within M1. Interestingly, this effect paralleled reduced motor task reaction time [53]. More recently, a double-blind study confirmed both the inhibitory effects on corticospinal excitability and the increased motor performance during low-intensity fTUS of M1. This study also found that MEP suppression was dose-dependent and accompanied by more effective short-interval intracortical inhibition (SICI) [50], a paired-pulse TMS measure reflecting GABA-A-ergic neurotransmission within M1 [55,56].

Similar to what has been observed in TMS studies, repetitive and patterned fTUS protocols have recently been conceived to induce long-lasting M1 plasticity changes. Repetitive fTUS (rTUS) for 15 min increased corticospinal excitability for up to 30 min after stimulation and reduced reaction time in a stop-signal task [51]. Similarly, an 80 s theta-burst patterned fTUS (tbTUS), which was derived from the original theta-burst stimulation protocol [57], produced consistent MEP facilitation for about 30 min. This novel neuromodulation paradigm also decreased SICI, increased ICF, and shortened movement time in a visuomotor task [52]. To the best of our knowledge, only one study tested the effects of low-intensity TUS on non-primary motor areas. Verhagen et al. (2019) applied 40 s fTUS on the supplementary motor area (SMA) in animals and found that activity coupling between the SMA and nearby areas increased for more than 1 h after stimulation, while the connectivity between the SMA and distant regions was reduced [58].

Concerning TUS effects on S1, some studies have suggested that stimulation exerts cortical excitation, while other lines of evidence support the opposite. Indeed, S1-fTUS in animals depolarized pyramidal neurons [59], increased S1 reactivity, modulated the spatial aspects of sensory receptive fields [60], induced action potentials in the targeted area [61], and determined hemodynamic changes, as measured by near-infrared spectroscopy, whose amplitude correlated with the peak intensity of the acoustic wave [62]. In line with the proposed excitatory effects of low-intensity TUS of S1, studies in humans have demonstrated that low-intensity fTUS elicits tactile sensations in the contralateral hand, evoking cortical potentials resembling somatosensory evoked potentials (SSEP) [63,64], and enhances sensory discrimination ability in a tactile vibration task through facilitatory mechanisms in cortical electroencephalography activity [65]. However, when analyzing the effect of low-intensity fTUS of S1 on SSEP, both animal and human studies demonstrated SSEP amplitude suppression during and after stimulation [66,67] and alterations in the spectral content and phase distribution of sensory-evoked brain oscillations [66,68]. Though seemingly at odds with neurophysiological data, these effects were, however, associated with improved somatosensory discrimination abilities [66]. The reasons for the opposed effect of S1-fTUS on SSEP and sensory performances are unclear. It might be due to methodological factors (e.g., different stimulating parameters between studies) and the different functional role of the putative neurons/circuits targeted by TUS in S1 on neurophysiological and behavioral measures.

As regards subcortical areas, the thalamus was the most widely targeted region in healthy conditions. In this setting, low-intensity fTUS has generally exerted inhibitory effects [8,14]. Indeed, various studies used SSEP amplitude as a proxy of sensory thalamic activity, and significant SSEP suppression was found with low-intensity TUS in swine [69], ovine [70], and rodents, with spatially and intensity-dependent specificity [13]. Consistent with animal data, low-intensity fTUS delivered unilaterally on the sensory thalamus inhibited the P14 SSEP component in a large, sham-controlled study in humans [5]. There was also an attenuation in alpha, beta, and time-locked gamma power and significant performance worsening on a discrimination task during stimulation [5]. In a different study, thermal pain sensitivity significantly decreased after 10 min low-intensity fTUS targeting the right anterior thalamus under MRI guidance [71]. Inhibitory fTUS effects have also been found when stimulating other subcortical areas, including the amygdala in healthy macaques [72] and the hippocampus in epilepsy mice [73], whereas excitatory effects have been reported when low-intensity fTUS was applied on the midbrain and periaqueductal grey in normal mice [74,75] and the substantia nigra (SN) and striatum in parkinsonian animal models (see Section 4). Finally, a recent study demonstrated that unfocused TUS on the superior colliculus increased trigeminal blink reflex excitability in healthy humans, possibly modulating inhibitory interneurons activity within this nucleus [6].

In summary, there is now significant evidence showing that low-intensity TUS modulates both cortical areas and deep structures, including the thalamus and brainstem and possibly the cerebellum [2,4,8,14]. The effects are highly variable and most likely depend on methodological factors that are currently not fully understood. However, the specific characteristics of the stimulated site may also affect the results. At the level of the sensorimotor cortex, inhibitory effects have been described in some cases [66,67], though most evidence shows an excitatory effect of TUS [59,60,61,62,63,64]. Conversely, the stimulation of deep brain areas has mainly inhibitory effects [2,4,8,14]. In addition to modifying neuronal excitability, low-intensity TUS seems to be able to modify the oscillatory activity of neuronal networks and, as recent evidence shows, also induce plasticity phenomena [51,52,68]. However, behavioral effects in terms of motor and sensory skills are less clear.

## 3. Possible Applications of TUS in Movement Disorders

Given the prominent role of M1 dysfunction in the pathophysiology of movement disorders [32,33,76,77], low-intensity TUS could be applied over M1 with the aim of ameliorating cortical neurophysiological abnormalities and motor performance. A cardinal neurophysiological feature of both parkinsonism and dystonia pathophysiology is the reduced inhibition at the M1 level, as demonstrated by paired-pulse TMS [32,78,79]. In PD, the impairment of GABA-A-ergic intracortical inhibition, as measured by SICI, is a very early and possibly prodromal alteration that is implicated as a pathophysiological mechanism underlying bradykinesia [76,77,78,80]. Patients with dystonia have decreased SICI both at rest and during movement initiation [81,82,83,84]. This mechanism is believed to contribute to altered M1 output, possibly resulting in unwanted muscle activation, co-contraction activity, and motor overflow phenomena in dystonia [85]. TUS could be an optimal neuromodulation tool to target mechanisms contributing to defective M1 inhibition. Indeed, M1-fTUS has been demonstrated to improve GABA-A-ergic intracortical neurotransmission in humans (increased SICI effectiveness [50]). A possible research approach could be to use M1-fTUS in PD and dystonia patients to evaluate whether reduced cortical inhibition can be restored and whether this determines the amelioration of PD motor symptoms or dystonic motor features (e.g., abnormal postures and co-contraction activity).

Another well-established neurophysiological alteration in PD and dystonia is abnormal M1 plasticity. In PD patients, M1 plasticity is impaired, according to findings from TMS-based protocols, and this impairment may contribute to movement dysfunction severity [20,76,86]. Conversely, experimental studies in dystonia have demonstrated prevailing facilitation of synaptic potentiation leading to homeostatic disruption [87]. M1 plasticity induced by paired associative stimulation (PAS) has been found to be abnormally enhanced in both focal hand and cervical dystonia patients [32,88,89]. Low-intensity fTUS protocols have been designed to induce long-term potentiation-like cortical plasticity in humans [51,52]. These paradigms could be tested in patients with PD and dystonia to verify whether TUS-induced plasticity mechanisms are altered like TMS-induced plasticity mechanisms in these conditions. It should also be evaluated whether this technique is able to obtain more reliable effects than those reported using TMS in movement disorders patients [90].

Concerning possible TUS effects on motor behavior, although human M1-TUS studies showed conflicting results regarding the direction of M1 excitability modulation (i.e., M1 excitability increase in [49] and decrease in [50,53]), all demonstrated improved motor function during or after low-intensity TUS, consistent with animal investigations [50,51,52,53]. Moreover, one recent study conducted in parkinsonian rats demonstrated encouraging results [31]. The authors delivered low-intensity fTUS over M1 (800 kHz, 100 Hz PRF, 10% DC, 6 s SD, 10 s ISI, 760 mW/cm^2^ I_SPPA_, 40 min/day) for 7 consecutive days and found improvements in locomotor and exploratory activity tasks as well as in bradykinesia and movement balance after 4–5 days of stimulation. After the entire treatment, there was also an increase in the number of c-Fos positive cells in M1 and in total superoxide dismutase and glutathione peroxidase activity in the striatum, suggesting that low-intensity TUS may have antioxidative effects in PD [31].

The SMA is another motor-related cortical area that could be targeted by low-intensity TUS. In PD, there is an increase in SMA-M1 coherence, and this neurophysiological alteration is hypothesized to reflect a compensatory mechanism of motor dysfunction [91,92,93]. A low-intensity fTUS study in healthy macaques demonstrated that functional connectivity between the SMA and M1 was enhanced after SMA stimulation [58]. Therefore, it would be interesting to apply low-intensity fTUS over the SMA in patients with PD and test whether SMA-M1 connectivity further increases and whether this change is associated with clinical motor scale improvement.

S1 neuromodulation could be another interesting field of TUS application that could be mostly applicable in dystonia. Sensory involvement is a clinical feature of dystonia patients [94], and neurophysiological evidence has demonstrated that defective somatosensory processing contributes to the network dysfunction underlying dystonia pathophysiology [32,95]. Patients with dystonia show abnormal tactile spatial and temporal discrimination [96,97], and these alterations are hypothesized to result from reduced inhibitory circuit excitability within S1 [98]. Importantly, the totality of S1-fTUS studies in animals and humans demonstrated beneficial effects on sensory functions during and after stimulation, including improved somatosensory discrimination abilities. Accordingly, there is enough rationale for delivering TUS over S1 in patients with dystonia to test whether stimulation restores impaired sensory task performance by modulating somatosensory processing at the S1 level. Modulation of somatosensory abilities could also be obtained by thalamic-fTUS, but negative effects (i.e., possible performance worsening) would be expected based on previous studies in animals and humans [5,71]. Therefore, we believe that applying thalamic-fTUS in dystonia would not be a particularly promising field of investigation.

Considering the high spatial resolution and penetration depth of TUS, another relevant opportunity in patients with movement disorders could be to stimulate deep brain regions, including the basal ganglia. A key neurophysiological feature in parkinsonism pathophysiology is the altered oscillatory activity in the basal ganglia-thalamo-cortical network. LFP recordings in the subthalamic nucleus (STN) of patients with PD have demonstrated exaggerated beta oscillations and reduced gamma activity power and burst rate, which correlated with cardinal motor symptom severity and contributed to movement force, velocity, and amplitude impairment [99,100,101,102,103]. Interestingly, a recent study in 1-methyl-4-phenyl-1,2,3,6-tetrahydropyridine (MPTP)-induced parkinsonian mice demonstrated that targeting the STN with fTUS (500 kHz, 1 kHz PRF, 5% DC, 50 ms SD, 1 s ISI, 5.1 W/cm^2^ I_SPPA_, 5 min total stimulation time) improved the typical pattern of altered oscillatory activity in PD, i.e., the stimulation significantly decreased mean beta power as well as the strength of beta-gamma and beta-ripple frequency phase-amplitude coupling early after stimulation [28]. Accordingly, it would be interesting to use STN-fTUS in PD patients to verify whether the stimulation ameliorates abnormal oscillations in the basal ganglia-thalamo-cortical loop and, in parallel, improves motor symptoms. In patients with dystonia, increased power of low-frequency oscillations (4–12 Hz) has been recorded in the globus pallidus internus (GPi), which correlated with abnormal EMG activity and dystonia clinical severity and is also involved in the modulation of dystonic contractions by sensory tricks [95,104,105,106]. In line with this evidence, deep brain stimulation (DBS) of the GPi, the most effective target to inhibit dystonic symptoms in patients [107], suppresses excessive low-frequency activity in the basal ganglia-thalamo-cortical loop and ameliorates M1 plasticity abnormalities [95]. Targeting the GPi with fTUS in patients with dystonia could therefore be a valid approach to improve GPi-related neurophysiological alterations and motor dysfunctions. However, the usefulness of GPi-fTUS in dystonia is dependent on the identification of appropriate stimulation parameters to induce inhibitory effects, ideally like those produced, albeit transiently, by FUS [17].

Importantly, TUS could be applied to the basal ganglia to improve bradykinesia in PD. Stimulation protocols that use repeated TUS applications over several weeks could be designed to test possible long-term changes in motor symptoms as well as the impact of chronic TUS on disease progression. These ideas are based on existing evidence in PD mice showing that 10 days of unfocused TUS treatment (1 MHz, continuous mode DC, 0.3 W/cm^2^ I_SPPA_, 5 min/day) restored locomotion activity and increased dopamine levels in the striatum, possibly due to neuroprotective effects and dopaminergic neurons regeneration [29]. In line with these data, a study conducted in a chronic PD mouse model showed motor function improvement following repeated applications of STN-fTUS (two sessions per week for 5 consecutive weeks, 3.8 MHz, 1 kHz PRF, 50% DC, 1 s SD, 4 s ISI, 430 mW/cm^2^ _ISPTA_), which was associated with increased expression of c-Fos in the STN, a marker of neuronal activity. In addition, the stimulation protocol suppressed neuroinflammation response in the SN and striatum by downregulating proinflammatory cytokines and signaling and reducing microglia and astrocyte activation [30]. Further corroborating the possible neuroprotective role of TUS administration in PD, a recent study found that 10 min of unfocused TUS applied five times every 24 h (1 MHz, 1 kHz PRF, 20% DC, ≈120 mW/cm^2^ I_SPTA_) reduced MPTP-induced neurotoxicity on dopaminergic neurons in mice. Indeed, in parallel to movement and balance dysfunction improvement, stimulation decreased the loss of tyrosine hydroxylase positive neurons in the SN pars compacta. Moreover, TUS attenuated MPTP-related decreased activity and improved intracellular oxidative stress and mitochondrial dysfunction [27].

The thalamus is another deep brain nucleus that could be targeted by fTUS. As detailed in Section 2, studies conducted in healthy animals and humans have demonstrated that fTUS exerts inhibitory effects on this brain area. Given the relevant role of the thalamus in tremors pathophysiology, not only in patients with essential tremor (ET) [108] but also in PD [37,109] and dystonia [110], it would be reasonable to test whether thalamic-fTUS is able to reduce tremor severity in patients during and/or after stimulation. In this regard, it is also worth mentioning that both DBS targeting the Vim nucleus of the thalamus and FUS thalamotomy are effective and FDA-approved treatments to suppress tremor in ET and tremor-dominant PD [17,111,112]. However, not all patients are suitable for these therapies. Vim-DBS and FUS are also limited by invasiveness and possible side effects [113,114]. The possible use of non-invasive neuromodulation techniques such as fTUS could provide relevant safety advantages. In our opinion, another possible field of TUS application in the future could be the target selection of movement disorder patients who are candidates for DBS or FUS. Once TUS effects on the STN, GPi, and thalamus are fully clarified, this non-invasive tool could be used as a pre-intervention procedure to test the effects produced by neuromodulation on each nucleus at the individual subject level when the most efficacious target is unclear (e.g., STN vs. GPi vs. thalamus in a tremor-dominant patient with PD or GPi vs. thalamus in a patient with dystonia and tremor).

The brainstem is another important brain area implicated in the pathophysiology of movement disorders. To date, it has been observed that unfocused TUS possibly modulates brainstem interneurons, although its effect can vary depending on the specific nucleus being stimulated (considering the polarity of the effects of various interneurons within the brainstem circuits) [6]. Moreover, the effects of brainstem-TUS may change according to the stimulation duration and other methodological factors. In both parkinsonism and dystonia, brainstem hyperexcitability has generally been found, as revealed by altered recovery cycle of the trigeminal blink reflex [32]. Particularly, brainstem hyperexcitability is considered a key mechanism underlying the orbicularis oculi muscles spasms in patients with blepharospasm [32]. Whenever TUS proves to be able to modulate brainstem excitability, it could, therefore, be used as a potential therapeutic tool in parkinsonism and dystonia.

Finally, the cerebellum is a node that plays a very important role in the pathophysiological network of movement disorders [108,115]. The cerebellum was early found to be a key region for action tremor generation [116]. Morphological and morphometric changes have been described in the Purkinje cells of patients with ET [117], and neuroimaging studies demonstrated structural cerebellar damage and connectivity alterations between the cerebellum and cortical areas, which are thought to be responsible for various motor and non-motor symptoms of ET [108]. More recently, it has been observed that the cerebellum also plays a key role in the pathophysiology of resting (parkinsonian) tremor [37] as well as in generating dystonic-like movements and postures [115]. In this regard, neurophysiological studies in dystonia consistently found cerebellar abnormalities, including impaired eye-blink classical conditioning, a simple form of motor learning that relies on olivopontocerebellar circuit activation [118,119,120], and reduced cerebellar-brain inhibition, a TMS measure reflecting the connectivity between the cerebellum and M1 [121]. There is also neuroimaging evidence of altered cerebellar activation in both sporadic and hereditary focal and generalized dystonia [115]. To date, different non-invasive neuromodulation techniques have been found to modulate cerebellar activity and connectivity [122,123,124].

**Figure 1:**
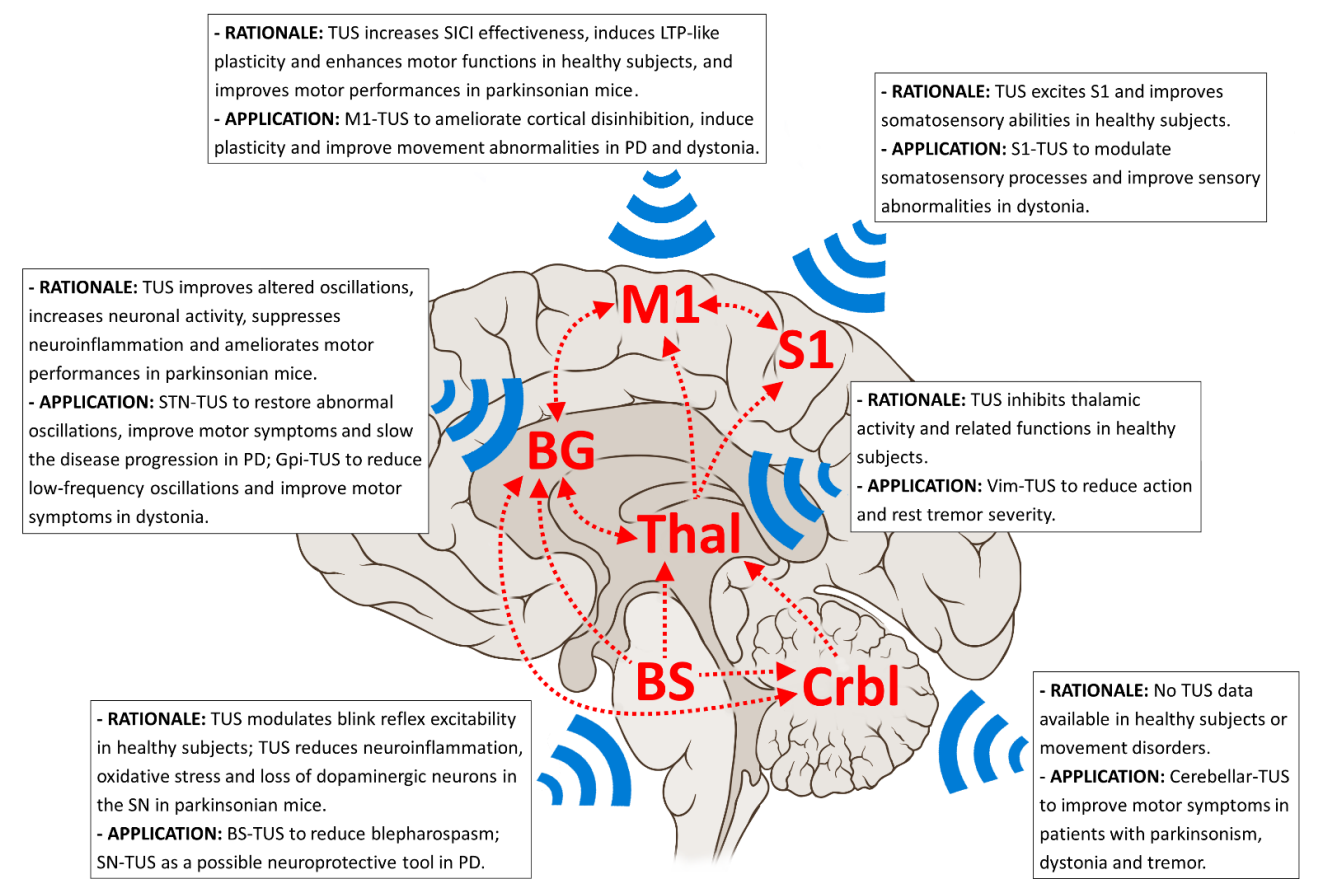
Most relevant nodes (red text) and pathways (red arrows) of the pathophysiological networks involved in Parkinson’s disease (PD), dystonia and tremor syndromes, including essential tremor (ET). The boxes summarize the rationale and proposed applications for possible neuromodulation using TUS in these movement disorders patients. BG: basal ganglia; BS: brainstem; Crbl: cerebellum; LTP: long-term potentiation; M1: primary motor cortex; S1: somatosensory motor cortex; SN: substantia nigra; STN: subthalamic nucleus; Thal: thalamus; Vim: ventral intermediate nucleus.

In our opinion, cerebellar TUS possibly represents a novel NIBS tool with multiple possible applications in patients with dystonia and tremor (Figure 1). Table 1 summarizes the available TUS studies in movement disorders.

## 4. Conclusions

Low-intensity TUS is a highly promising NIBS tool. In contrast to TMS and TES, the ultrasound beam can reach small and deep brain areas (including the basal ganglia, thalamus, brainstem, and cerebellum) that are crucial to the pathophysiology of parkinsonism, dystonia, and tremor. Moreover, TUS mechanisms of action largely differ from those of previous non-invasive brain stimulation techniques. Biomechanical and thermal modifications in the neuronal membrane might determine different, and potentially greater, effects than those reported using TMS or TES on cortical areas in movement disorder patients [90]. There are currently important limitations affecting TUS applications in movement disorders. First, TUS neurophysiological effects on healthy subjects are scarce and sometimes conflicting (i.e., excitatory vs. inhibitory effects). This is mainly due to the lack of homogeneity in the stimulating parameters used across the various studies, including the intensity and fundamental frequency of stimulation, duty cycle, and sonication duration. Moreover, it is likely that focused and unfocused TUS devices operate through partially different mechanisms of action, thus determining different effects [49,50]. Accordingly, methodological studies on large samples are needed that systematically examine TUS effects on cortical and subcortical areas according to the stimulation parameters used. A further relevant limitation is that only five studies have been conducted on animal models of movement disorders, and all involved parkinsonian mice. TUS has never been used on animal models of parkinsonism, dystonia, and tremor. More importantly, no study has involved movement disorder patients. Due to neuroanatomical and neurophysiological differences between mice and humans, it is possible that TUS responses in PD patients differ from those reported in parkinsonian animals. Additionally, beneficial effects could not be observed in patients because of suboptimal TUS stimulation parameters. Furthermore, concerning the various experimental approaches that we proposed in parkinsonism, dystonia, and tremor, TUS may not necessarily modulate the activity of the different targets in the expected direction. For instance, since no previous study targeted the GPi, it is difficult to predict whether TUS would exert positive clinical effects as hypothesized. In addition, although the brainstem and cerebellum are both crucial nodes in movement disorders, there are only few or no data on the TUS effects on cerebellar activity. Therefore, future studies are needed in healthy humans and in patients with movement disorders to better delineate the neuromodulator role of TUS in these conditions.

## Figures and Tables

**Table 1:** Available TUS studies in movement disorders.

Reference	Study Subjects	Target area	TUS Parameters	Stimulation Protocol	Key Findings	Significance
Zhou et al., 2019 [31]	Parkinsonian rats	M1	800 kHz, 100 Hz PRF, 10% DC, 6 s SD, 10 s ISI, 760 mW/cm^2^ I_SPPA_	40 min/day for 7 days	- Improved locomotor activity, movement balance and bradykinesia - Increased c-Fos + cells in M1 and total SOD and GPx activity in the striatum	M1-TUS ameliorates motor symptoms and exerts antioxidative effects in PD
Wang et al., 2020 [28]	Parkinsonian mice	STN	500 kHz, 1 kHz PRF, 5% DC, 50 ms SD, 1 s ISI, 5.1 W/cm^2^ I_SPPA_	5 min total stimulation time	- Decreased beta power - Decreased beta-gamma and beta-ripple frequency PAC	STN-TUS improves the typical pattern of altered oscillatory activity in PD
Xu et al., 2020 [29]	Parkinsonian mice	Whole brain	1 MHz, continuous mode DC, 0.3 W/cm^2^ I_SPPA_ (unfocused TUS)	5 min/day for 10 days	- Improved locomotion - Increased dopamine levels in the striatum	TUS ameliorates motor symptoms and may induce dopaminergic neurons regeneration
Zhou et al., 2021 [30]	Parkinsonian mice	STN	3.8 MHz, 1 kHz PRF, 50% DC, 1 s SD, 4 s ISI, 430 mW/cm^2^ I_SPTA_	2 sessions per week for 5 weeks	- Improved movement coordination, balance, and bradykinesia - Increased c-Fos expression in the STN - Downregulation of proinflammatory signaling and reduced activation of microglia and astrocytes in the SN and the striatum	STN-TUS improves motor functions and suppresses the neuroinflammation response in basal ganglia in PD
Chen et al., 2021 [27]	Parkinsonian mice	Whole brain	1 MHz, 1 kHz PRF, 20% DC, ≈120 mW/cm^2^ I_SPTA_ (unfocused TUS)	10 min/day for 5 days	- Improved movement and balance - Decreased loss of TH + neurons in the SN - Improved intracellular oxidative stress and mitochondrial dysfunction	TUS improves motor dysfunctions and may have neuroprotective effects in PD

DC: duty cycle; GPx: glutathione peroxidase; ISI: interstimulus interval; I_SPPA_: spatial-peak pulse average; I_SPTA_: spatial-peak temporal average; M1: primary motor cortex; PAC: phase-amplitude coupling; PD: Parkinson’s disease; PRF: pulse repetition frequency; SD: sonication duration; SOD: superoxide dismutase; SN: substantia nigra; STN: subthalamic nucleus; TH: tyrosine hydroxylase.
